# Human intestinal organoids express histo-blood group antigens, bind norovirus VLPs, and support limited norovirus replication

**DOI:** 10.1038/s41598-017-12736-2

**Published:** 2017-10-03

**Authors:** Dongsheng Zhang, Ming Tan, Weiming Zhong, Ming Xia, Pengwei Huang, Xi Jiang

**Affiliations:** 1 Division of Infectious Diseases, Cincinnati Children’s Hospital Medical Center, Cincinnati, OH USA; 2 Department of Pediatrics, University of Cincinnati College of Medicine, Cincinnati, OH USA

## Abstract

Through pluripotent stem cell (PSC) technology, human intestinal organoids (HIOs) with remarkably similarity to the fetal intestine in cellular composition, architecture, and absorptive/secretory functions have been successfully developed, providing a useful *in vitro* model system to study the structure and function of human congenital gut and intestinally related diseases. We report here the usefulness of HIOs as a model system to study intestinal carbohydrate expression, virus-host interaction, and replication of human noroviruses (huNoVs). We found that fully developed HIOs express effectively various types 1 and 2 HBGAs, including Lewis, secretor, and nonsecretor antigens, distributing on the glycocalyx. Selected huNoV-like particles (VLPs) bound the glycocalyx of HIOs with matched HBGA phenotypes. Using GII.4 huNoV positive stool filtrates, we demonstrated limited huNoV replication in HIOs with corresponding HBGAs through detection of viral RNAs by RT-PCR and capsid antigens by immunostaining methods. Our data suggested that, after further improvements, HIOs can be a useful model to study intestinal glycan expression, huNoV-intestine interaction, and huNoV infection in the intestine.

## Introduction

The recent advancements of human pluripotent stem cell (PSC) technologies provide a powerful *in vitro* or *ex vivo* system to study human intestinal biology and related illnesses^1–3^. PSCs are master cells that are able to make cells from all three basic body layers and therefore can potentially differentiate into any cell or tissue of human body. For example, three dimensional intestinal tissues of humans, referred as human intestinal organoids (HIOs), have been developed *in vitro* through directed differentiation of human PSCs using several specific grow factors, including activin A for endoderm differentiation, fibroblast growth factor 4 (FGF4) and Wnt3a for hindgut differentiation, as well as R-spondin 1, noggin, and epidermal growth factor (EGF) for intestinal organoid differentiation^1–3^. Fully developed HIOs are composed of an epithelial layer enclosed by a mesenchyme. The HIO epithelium contains all major intestinal cell types, including absorptive enterocytes, the three secretory lineages of goblet cells, enteroendocrine cells, and Paneth cells, and crypt-like proliferative zones that are composed of intestinal stem cells. In other words, well differentiated HIOs exhibit structural and functional properties of human intestine^1–3^, offering an excellent *ex vivo* model system to study the biology of human congenital gut and intestinally related diseases.

Human noroviruses (huNoVs), a group of single stranded, positive sense RNA viruses constituting the *Norovirus* genus of family *Caliciviridae*, cause epidemic acute gastroenteritis in humans in all age groups with higher prevalence in children and the elderlies, leading to significant morbidity and mortality^4,5^. United States CDC estimated that huNoVs cause approximately 21 million infections in the USA and 218,000 deaths worldwide annually^4^. HuNoVs are highly contagious and are transmitted by contaminated food and water and by person-to-person contacts, commonly leading to large community outbreaks. Thus huNoVs remain a serious public health concern.

HuNoVs have been shown to infect enterocytes of intestine via attachment to histo-blood group antigens (HBGAs) on the mucosal surface or glycocalyx layer of intestine^6,7^. HBGAs are complex, fucose-containing carbohydrates that distribute abundantly on the mucosal surface of gastrointestinal tract, serving as the attachment factor to initiate huNoV infections^8–10^. Significant advancement of knowledge, including those learned from human challenge studies^11–14^ and huNoV outbreak investigations^15,16^, indicates that HBGAs are important receptors and host susceptibility factors to huNoV infection.

HuNoVs remain difficult to study due to the lack of a conventional culture system. Many human and animal cell lines under variable cultivation conditions have been tested by different laboratories for huNoV replication without success. A 3-dimensional cell culture system^17^ was reported in supporting huNoV replication, but appears not to be reproducible in other laboratories. In addition, a B cell line was developed to culture huNoVs with limited efficiency^18^, which however has not yet widely proved. Until recently solid data of huNoV replication *in vitro* have been reported using human intestinal crypt stem cell-derived enteroids^19^. Although further improvements for higher viral replication efficiency are needed, this human intestinal enteroid (HIE) culture system provides a valuable *in vitro* model for future mechanistic study of huNoV infection and pathogenesis. In this current study, using similar procedures of *in vitro* differentiation and cultivation of HIEs, we established HIOs and characterized differentiated HIOs for their HBGA expression and distribution, specific interactions with huNoV VLPs, and huNoV replication. We found that HIOs expressed both type 1 and 2 HBGAs, bound huNoV capsids with corresponding HBGA binding profiles, and supported limited huNoV replication. Our data indicated a potential of HIOs as a useful model to explore intestinal carbohydrate expression, huNoV-intestine interaction, and huNoV infection in the intestine.

## Results

### Development of HIOs

**Figure 1:**
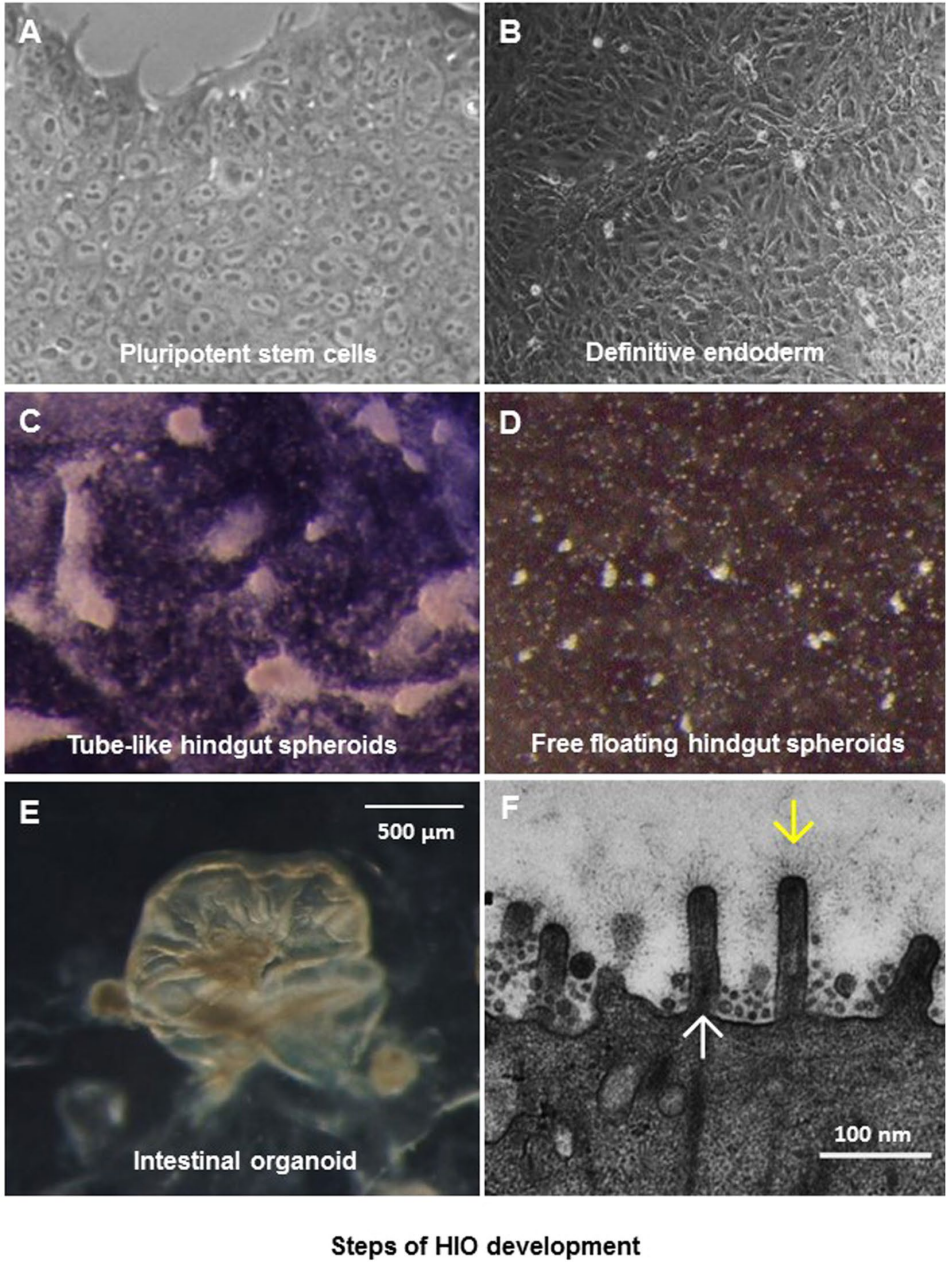
Different stages of human intestinal organoid (HIO) development. (**A**) Morphology of a single layer culture of human pluripotent stem cells (PSCs). (**B**) Morphology of PSC-derived definitive endoderm. (**C** and **D**) Morphologies of PSC-derived hindgut spheroids, including tube-like (**C**) and free floating (**D**) hindgut spheroids. (**E**) Morphology of a fully developed HIO. (**F**) An EM micrograph showing typical microvilli (upward arrow) with carbohydrates (downward arrow) on the surface of a fully developed HIO.

Our HIO development started with human PSCs (Fig. 1A). As expected, the stem cells first differentiated to definitive endoderm (Fig. 1B) under induction with Activin A. The endoderm developed further into hindgut spheroids with induction of fibroblast growth factor 4 (FGF4) and Wnt3a. Culture at this stage contains tube-like spheroids (Fig. 1C) and free floating spheroids (Fig. 1D). The hindgut spheroids developed finally into HIOs (Fig. 1E) with typical intestinal morphogenesis, including polarized, columnar epithelia featured with brush borders and microvilli, as shown by EM micrographs (Fig. 1F). A layer of glycocalyx consisting of various carbohydrates was visualized on the surface of the microvilli covering the epithelium.

### HIOs containing typical intestinal epithelial cells

**Figure 2:**
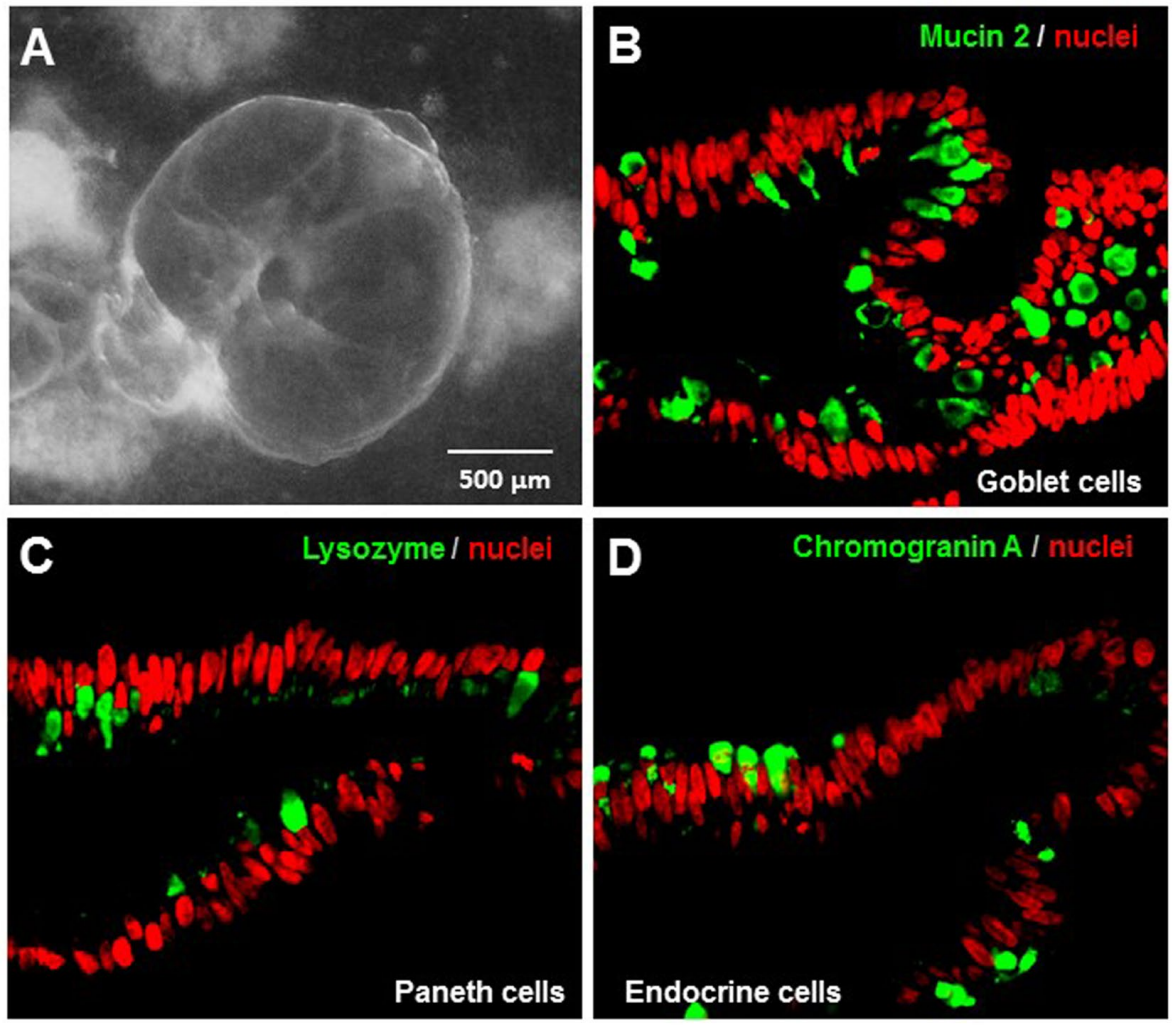
Cellular composition of human intestinal organoids (HIOs). (**A**) Morphology of a fully developed HIO. (**B** to **D**) Goblet cells (**B**), Paneth cells (**C**), and enteroendocrine cells (**D**) of HIOs shown by monoclonal antibodies specific to the biomarkers of mucin 2 (goblet cells), lysozyme (Paneth cells), and chromogranin A (enteroendocrine cells), respectively, through immunofluorescent microscopy. All the three cell biomarkers are shown in green. Nuclei of the epithelial cells are stained by TO-PRO-3 stain in red.

The fully developed HIOs (Fig. 2A) were further characterized for their cell type composition of the epithelia using Mabs against specific intestinal cell biomarkers. All intestinal cell types, including goblet cells as shown by mucin 2 (Fig. 2B), Paneth cells by lysozyme (Fig. 2C), and enteroendocrine cells by chromogranin A (Fig. 2D) were identified among the enterocytes that constitute the majority of the HIO epithelia as shown nuclei stained by TO-PRO-3 stain (Fig. 2B–D, red color). The above data collectively indicated that the HIOs were fully developed and ready for further characterization of HBGA expression, huNoV interaction, and huNoV replication (below).

### Genotyping the secretor vs. nonsecretor status of PSC lines

**Figure 3:**
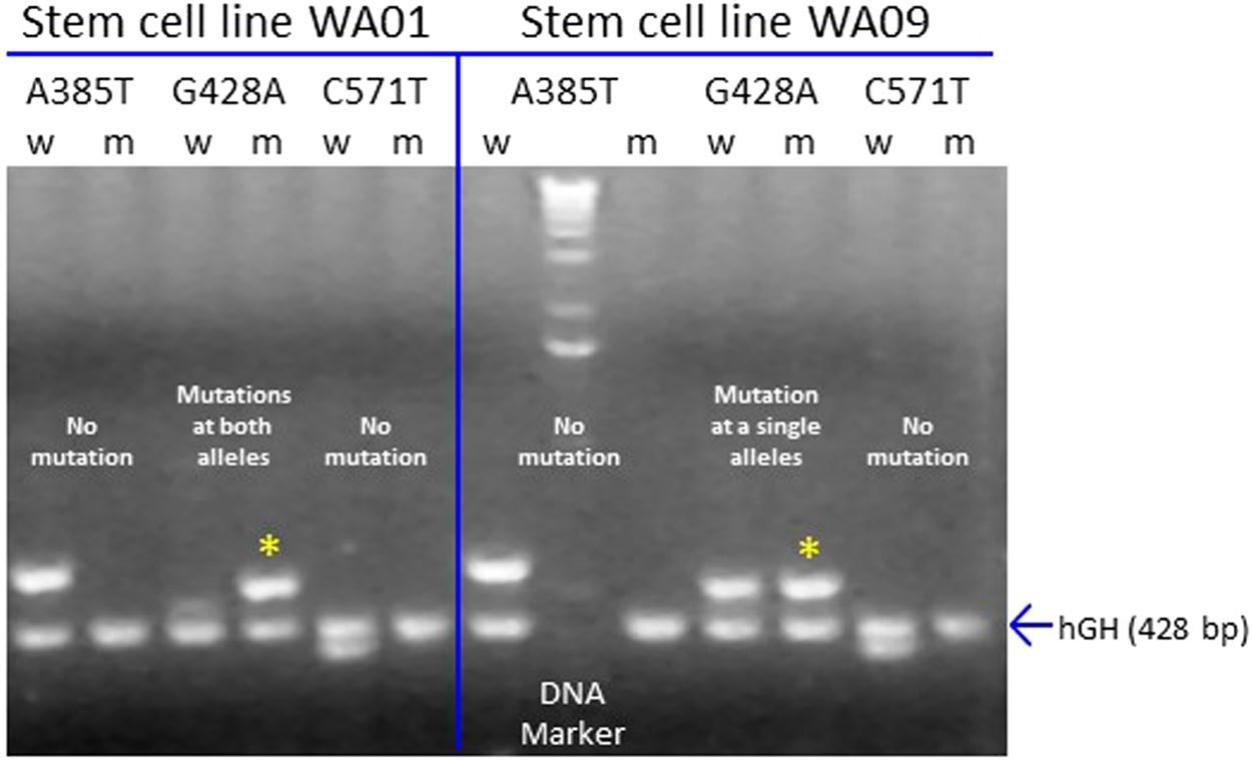
Genotyping α 1,2-fucosyltransferase (FUT2)-encoding gene (*FUT2*) in two human stem cell lines, WA09 and WA01. Three predominant nonsense mutations (A358T, G428A, and C571T) in the *FUT2* gene were tested by sequence-specific primer-directed PCR (SSP-PCR) using primers specific three mutations, respectively. PCR products for both wild type (w) and mutant (m) genes that are 575/576, 529/530, and 389/390 bp respectively, are shown. The detected G428 mutations in the *FUT2* gene in both the stem cell lines are indicated by star symbols. The gene-encoding the human growth hormone (hGH) was served as an internal control for both DNAs and PCRs with an expected PCR product of 428 bp. This is a full-length gel.

Enzyme FUT2 catalyzes the formation of H antigen that is the key determinant of secretor phenotypes. Mutations in the *FUT2* gene lead to functional loss of the FUT2 enzyme, resulting in nonsecretor status. Three nonsense mutations (A358T, G428A, and C571T) that disrupt the normal translation of a functional enzyme are commonly seen in humans^20^. They were mapped by sequence-specific primer-directed PCRs (SSP-PCRs) using specific primers (Table 1) described in Materials and Methods. Our PCR results (Fig. 3) showed that stem cell line WA09 has a G428A mutation in one of the two *FUT2* alleles and thus should be heterozygous dominant, being able to express a functional FUT2 enzyme. Therefore, WA09 will develop into an HIO line with secretor phenotypes. By contrast, the stem cell line WA01 has the G428A mutations in both *FUT2* alleles and thus cannot express a functional FUT2 enzyme. Thus, WA01 will develop into an HIO line with nonsecretor status.

**Table 1:** Primer sequences and the expected PCR product sizes.

Name	Note	Sequences (5′ to 3′)	Product size
P1773-w385A	FUT2 wild-type forward	AGGAGGAATACCGCCACAT	575 bp
P1774-m385T	FUT2 mutated forward	GAGGAGGAATACCGCCACT	576 bp
P1775-w428G	FUT2 wild-type forward	GCTACCCCTGCTCCTGG	529 bp
P1776-m428A	FUT2 mutated forward	CGGCTACCCCTGCTCCTA	530 bp
P1777-w571C	FUT2 wild-type forward	TAGGGGTC CATGTTCGCC	389 bp
P1778-m571T	FUT2 mutated forward	GTAGGGGTCCATGTTCGCT	390 bp
P1772-fut2 rev	FUT2 reverse primer	GGCTGCCTCTGGCTTAAAG	/
P1779-hGH	hGH forward	GCCTTCCCAACCATTCCCTT	428 bp
P1780-hGH rev	hGH reverse	TCACGGATTTCTGTTGTGTTTC	/

### HBGA phenotyping of HIOs

**Figure 4:**
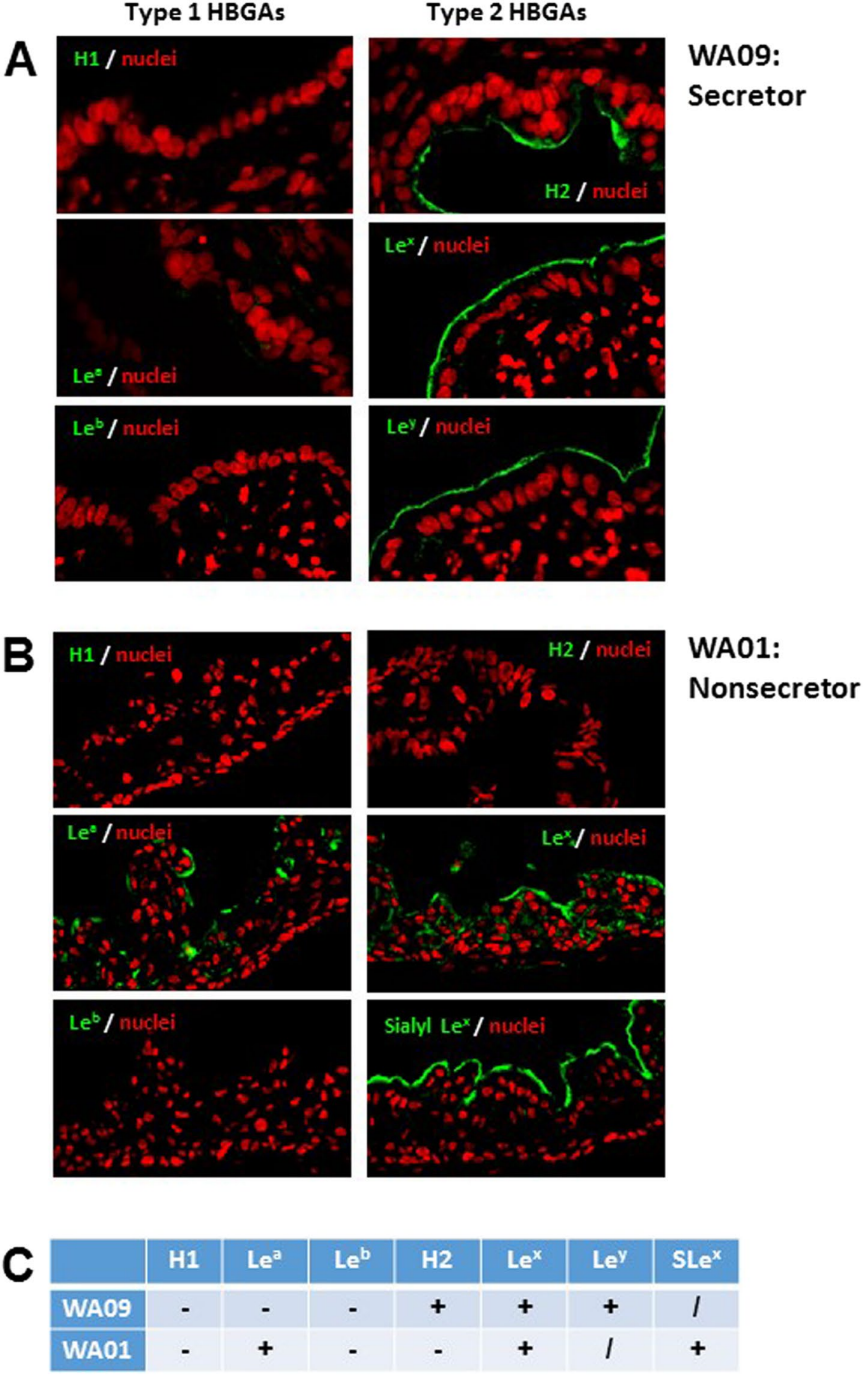
HBGA phenotyping of human intestinal organoids (HIOs). The two selected HIO lines, WA09 and WA01, with known genotypes of the *FUT2* gene were examined for their expression profiles of various HBGA antigens through immunofluorescent microscopy using monoclonal antibodies (Mabs) specific to each of three type 1 (H1, Le^a^ and Le^b^) and four type 2 (H2, Le^x^, sialyl Le^x^, and Le^y^) antigens. (**A**) The secretor HIO WA09 expresses type 2 (right panel: H2, Le^x^, and Le^y^) but not type 1 (left panel: H1, Le^a^ and Le^b^) antigens. (**B**) The nonsecretor HIO WA01 expresses type 1 (left panel: Le^a^) and type 2 (right panel: Le^x^ and sialyl Le^x^) nonsecretor antigens but not the secretor antigens (H1 and Le^b^). HBGA signals are shown in green, while nuclei of the epithelia were shown in red. (**C**) Summary of the HBGA phenotypes of the two HIOs. (+), positive; (−), negative, and (/) not tested.

The expression of different HBGAs by WA09 and WA01 HIOs were further examined through immunofluorescent microscopy using specific Mabs. We found that HIO WA09 expressed secretor antigens of H type 2 and Le^y^. It also expressed Le^x^ antigen, the catalyzed product of the α 1,3/4 fucosyltransferase (FUT3) (Fig. 4A, left panel, and Fig. 4C). Thus, WA09 should be categorized as secretor positive HIO based on the two type 2 secretor products (H type 2 and Le^y^ antigen), even though it did not produce type 1 HBGA secretor products (Fig. 4A, right panel). By contrast, HIO WA01 expressed nonsecretor antigens, including type 1 Le^a^ antigen, as well as type 2 Le^x^ and Sialyl Le^x^ antigens, but did not expressed any secretor antigen (Fig. 4B,C). These data confirmed the genotyping results and provided useful information for further studies of huNoV-host interaction and huNoV replication/infection using these two HIO lines. It was noted that the vast majority of HBGA signals distributed at the glycocalyx, corresponding to the outermost surfaces of the epithelia (Fig. 4A,B).

### HuNoV VLPs Binding HIOs with matched HBGA phenotypes

**Figure 5:**
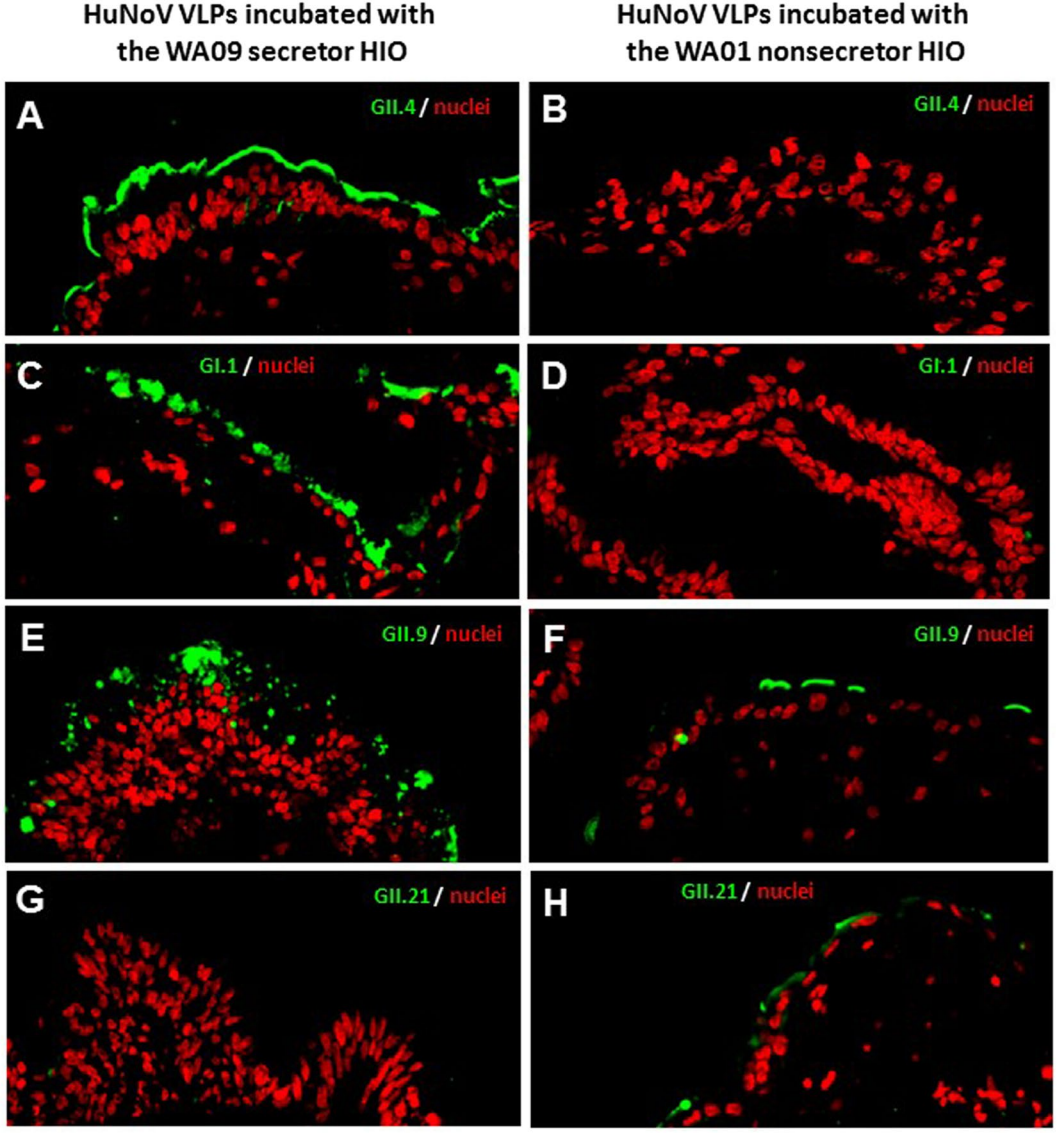
HuNoV VLPs bound human intestinal organoids (HIOs) with matched HBGA phenotypes. The binding of various huNoV VLPs to the secretor WA09 and the nonsecretor WA01 HIOs were shown by immunofluorescent microscopy. This was achieved by detecting the bound VLPs on thin sections of the HIOs using polyclonal antibodies specific to huNoVs, followed by a fluorescently labelled secondary antibody. (**A**–**D**) GII.4 VA387 (**A** and **B**) and GI.1 Norwalk virus (**C** and **D**) are secretor binders and their VLPs bound the glycocalyx of the WA09 (**A** and **C**), but not the WA01 HIO (**B** and **D**). (**E** and **F**) GII.9 VA207 binds both secretor and nonsecretor HBGAs and its VLPs bound both WA09 and WA01 HIOs. (**G** and **H**) GII.21 OIF is a nonsecretor binder and OIF VLP bound the WA01 nonsecretor HIO (**H**), but not the WA09 secretor HIO (**G**). The bound huNoV VLPs are shown in green, while the nuclei of the HIO epithelia are shown in red.

Previous studies showed that huNoVs bind different HBGAs in a strain-specific manner^8–10^. In this study, we found that both GII.4 VA387 and GI.1 Norwalk virus VLPs, which recognize most secretor, but not nonsecretor HBGAs^21^, bound the glycocalyx of the WA09 but not the WA01 HIOs (Fig. 5A–D). In addition, GII.9 huNoV VLPs that recognize Le^x^ and Le^y^ antigens^21,22^ bound both HIOs, but the binding signals to WA09 that expressed both Le^x^ and Le^y^ antigens were clearly stronger than those to WA01 that expressed only Le^x^ antigen (Fig. 5E,F). Furthermore, the GII.21 huNoV VLPs that recognize Le^a^ antigen only^23^ bound the Le^a^ positive WA01 but not the Le^a^ negative WA09 HIO (Fig. 5G,H).

### Limited huNoV replication in HIOs

**Figure 6:**
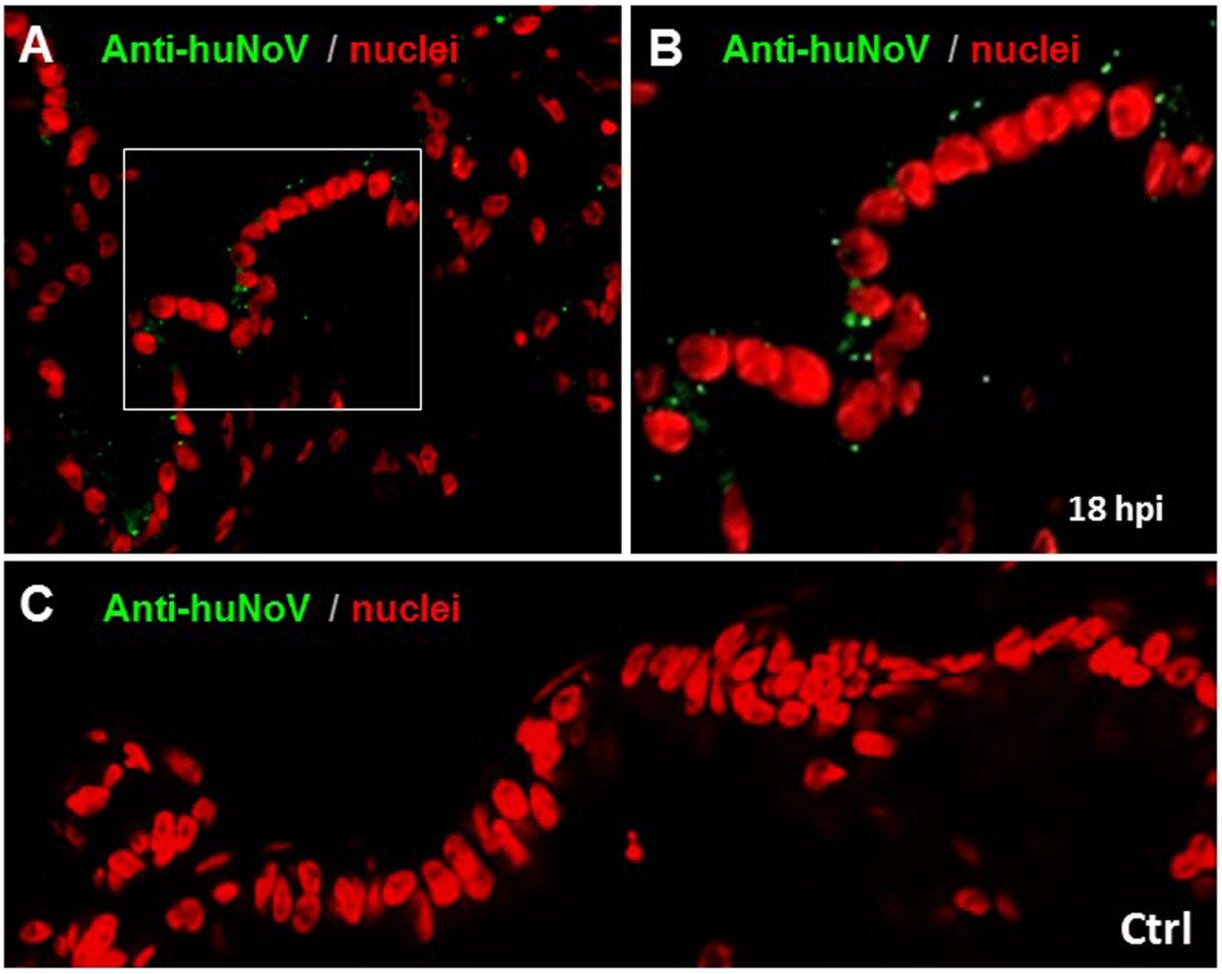
Detection of limited GII.4 huNoV replication in secretor human intestinal organoids (HIOs) by immunofluorescent staining of huNoV capsid antigens. HuNoV inoculated WA09 HIOs were harvested at 18 hours after removal of the huNoV inoculum. Thin sections of the HIOs were stained with polyclonal antibody against GII.4 huNoV VLPs, followed by incubation with fluorescently labeled secondary antibody. Scattered green dots locating in cytoplasm of epithelium cells (**A** and **B**) were detected in the inoculated HIOs but not in the mock inoculated HIO control using inactivated huNoVs (**C**). (**B**) is an enlargement of the framed region in (**A**). The replicated huNoV capsid antigens are shown in green, while the nuclei of the HIO epithelia are shown in red.

The above observed features prompted us to test whether HIOs support huNoV replication and the WA09 secretor HIO was selected for the study. Stool filtrates with confirmed GII.4 huNoVs^14^ were used to infect WA09 HIO. At 18 hours after removal of the huNoV inoculum huNoV capsid antigens were detected in the cytoplasm near the surface in some epithelial cells of the HIOs using polyclonal antibodies specific to GII.4 huNoV, while the negative control using inactivated huNoVs by boiling for 10 min did not show similar signals (Fig. 6). It was noted that, unlike huNoV VLPs that bound mainly to the mucosal surface glycocalyx, the replicating huNoV signals were detected as scattered dots in the cytoplasm of the infected enterocytes by both immunofluorescent/confocal microscopy (Fig. 6).

**Figure 7:**
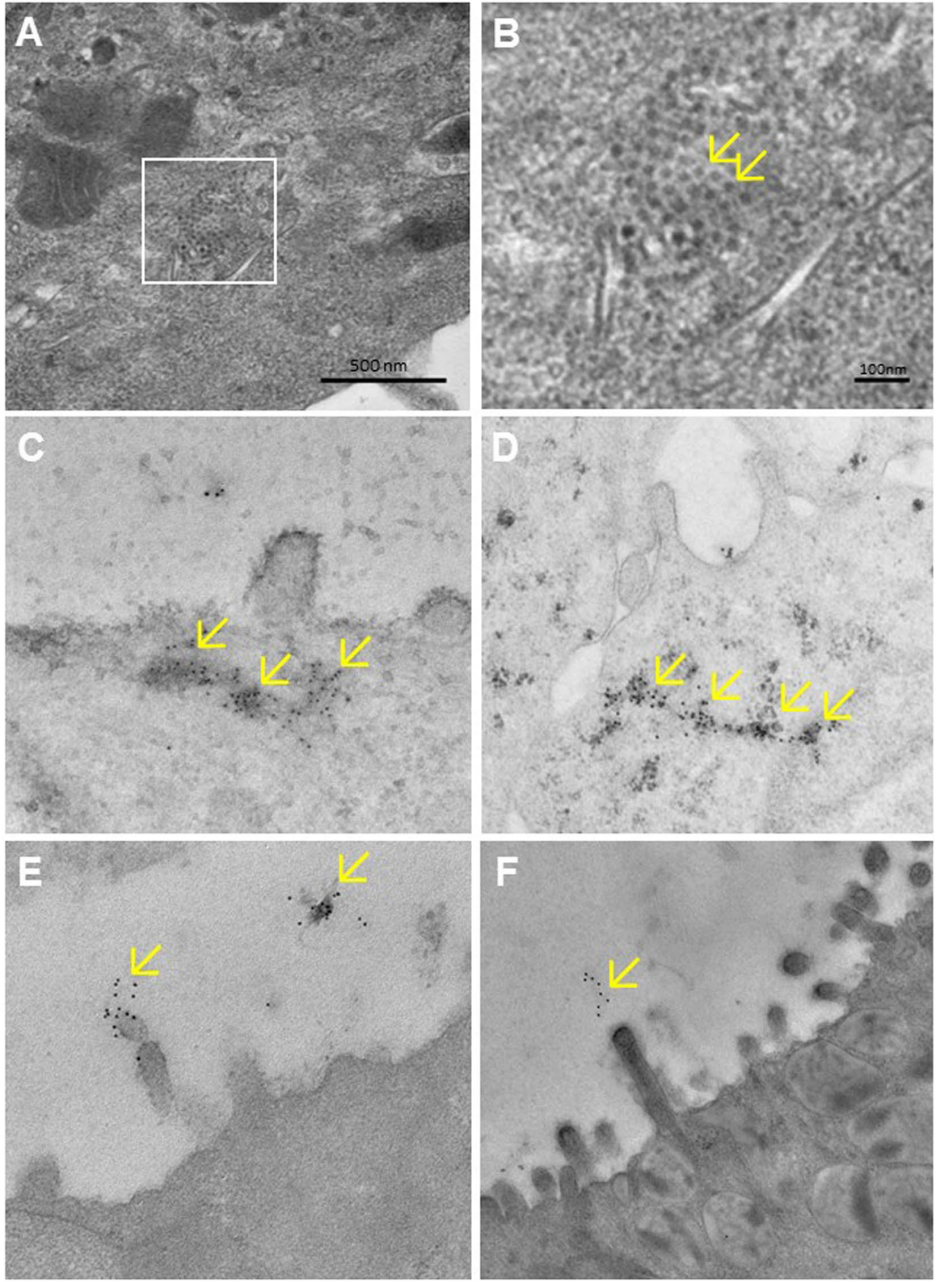
Detection of limited huNoV replication in human intestinal organoids (HIOs) by electron microscopy (EM) at 18 hours after removal of the huNoV inoculum. (**A** and **B**) HuNoV-like particles were seen in an inclusion body by negative staining EM (**A**) (framed area). The framed region of (**A**) is enlarged in (**B**) with arrows pointing to NoV-like particles. (**C**–**F**) HIO sections were stained with immunogold-labeled antibodies specific to GII.4 huNoV capsid antigen. Scattered immunogold signals (arrows) cluster in intracellular vacuoles (**C** and **D**) and on the glycocalyx layer of microvilli (**E** and **F**).

Replicating huNoVs in WA09 HIO were also detected by EM. HuNoV-like particles were seen in potential inclusion bodies in the cytosol of some enterocytes at 18 hours after removal of the huNoV inoculum (Fig. 7A,B), corresponding to the dot signals detected by immunofluorescent microscopy (Fig. 6). Accordingly, immunogold staining using GII.4 huNoV-specific antibody demonstrated the presence of huNoV antigens in the intracellular vacuoles at the same timing (Fig. 7C,D). In addition, we noted that some immunogold particles clustered on the glycocalyx of microvilli, consistent with the observation by immunofluorescent microscopy that some dot signals located near the surface of the HIO enterocytes. This may be due to the interactions between huNoVs and HBGA carbohydrates in the glycocalyx (Fig. 7E,F). These huNoVs or huNoV signals were not present in the negative control using inactivated huNoVs by boiling for 10 min (data not shown).

**Figure 8:**
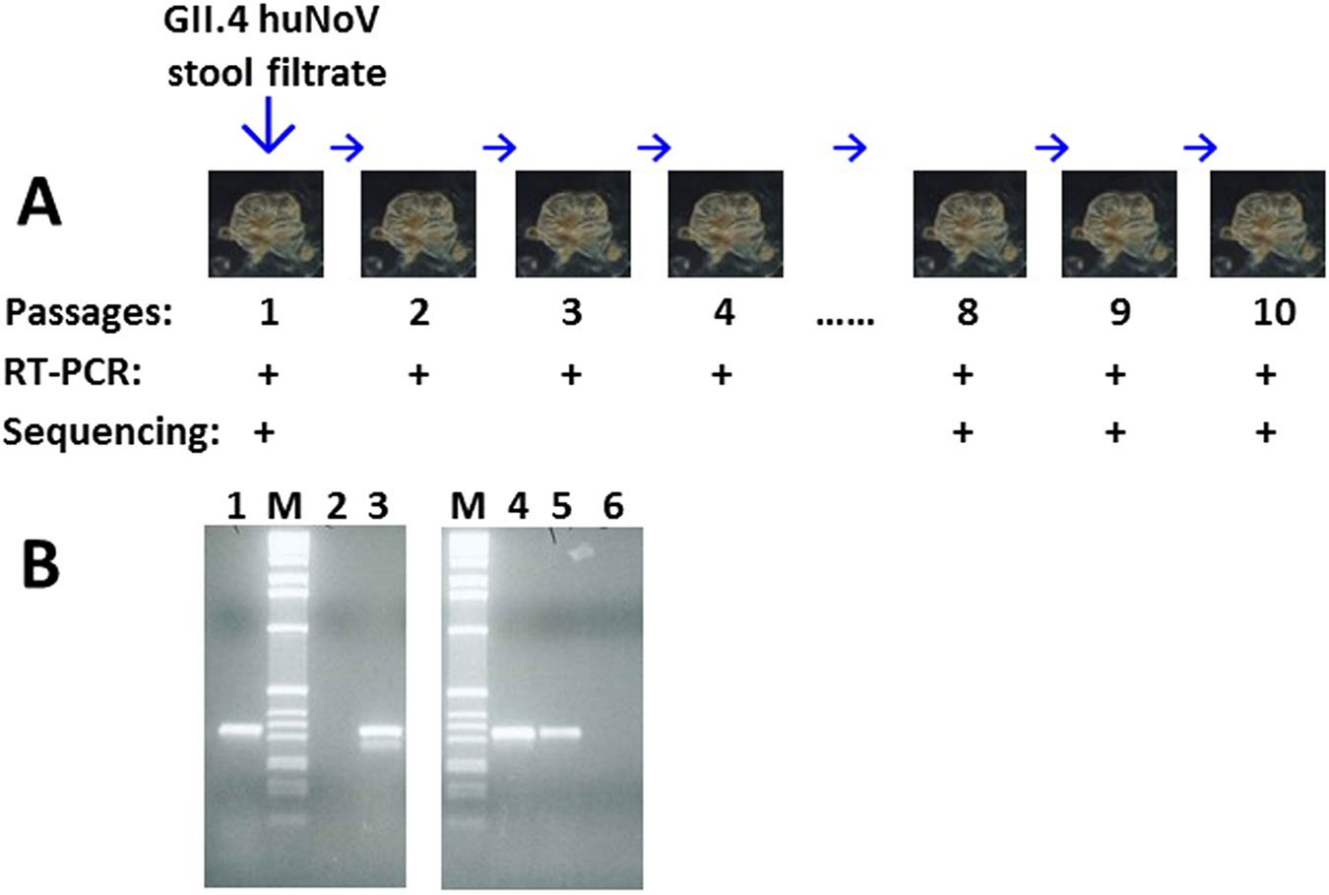
Detection of replicated huNoV RNAs by RT-PCR following multiple passages of human intestinal organoids (HIOs). (**A**) HIOs were first inoculated by GII.4 huNoV stool filtrate. The inoculated HIOs were harvested on day 4 post inoculation to make HIO homogenization supernatant for RT-PCR detection and inoculation of fresh HIOs. This process was repeated for 10 times (passages). GII.4 huNoV RNAs were detected positively by RT-PCR in all seven indicated passages, while 5^th^ to 7^th^ passages were not tested. Sequencing of the PCR products from the original huNoV inoculum and the last three passages showed identical GII.4 huNoV. (**B**) Representative PCR results showing positively detected huNoV RNAs. M, DNA markers; lane 1, positive control with viral samples from stool sample; lanes 2 and 6, negative controls using uninfected HIO homogenization supernatant; lanes 3, 4, and 5, PCR products (310 bp) from passages 1, 9 and 10, respectively. Both graphs are from full-length gels.

HuNoV replication in HIOs was also supported by multiple passages of GII.4 huNoV inoculums. For each passage, the inoculated HIOs were homogenized to release viruses and the supernatant of homogenized HIOs was then inoculated to a fresh HIO culture. This was repeated for 10 times (Fig. 8). GII.4 huNoVs were detected in all 7 RT-PCRs among the HIO passages; while RT-PCR were skipped for other three passages due to smaller sizes of host HIOs used. Sequencing of the huNoV PCR products from the original inoculum and the last three passages showed the identical GII.4 virus, indicating that these huNoV were descendants of the GII.4 huNoVs from the original inoculum (Fig. 8).

## Discussion

With help from the Pluripotent Stem Cell Facility (PSCF) at our institution (https://research.cchmc.org/stemcell/overview), we have successfully established two HIO lines in our laboratory that represent secretor and non-secretor populations of humans, respectively. The two HIOs have been characterized thoroughly for their features in HBGA expressions and specific interactions with huNoV VLPs. In addition, we have observed limited huNoV infection and replication in the HIOs. Thus, our study provides evidence that HIOs may be used as a useful tool to study the dynamic expression and distribution of intestinal carbohydrates, huNoV-enterocytes interaction, and huNoV infection in the intestine.

HuNoVs have been shown to recognize HBGAs as host attachment factors for infection^11,13,14^. Matched HBGA types of HIEs were also shown to be required to support huNoV replication *in vitro* ^19^. To obtain HIOs with right HBGA phenotypes as a prerequisite for huNoV interaction and infection, we first screened several available stem cell lines for a functional and a mutant *FUT2* gene encoding the FUT2, the key enzyme that catalyzes the production of H antigen to initiate the secretor pathway^24,25^. Stem cell line WA09 was found to have a functional *FUT2* gene, while WA01 does not. Thus, the two stem cell lines that represent human secretor (WA09) and nonsecretor (WA01) populations, respectively, were selected for HIO development.

To further define the detailed HBGA phenotypes, we determined on the expression of both type 1 and 2 H and Lewis antigens of the two HIOs. Corresponding to its nonsecretor genotype, the WA01 HIO expressed typical Le^a^, Le^x^, and sialyl Le^x^ antigens without any H-related antigen. By contrast, the secretor HIO WA09 expressed only H-related antigens, including H2 and Le^y^ antigens. Consistent with these defined HBGA phenotypes, the secretor HBGA-binding VLPs of GII.4 VA387 and GI.1 NV^21^ bound only WA09 HIOs; the nonsecretor-binding VLPs of GII.21 OIF^23^ bound only HIO WA01; while VLPs of VA207^22^ bound both HIOs. These huNoV VLP-HIO binding outcomes were consistent with the previously observed VLP-HBGA binding profiles, which are strain specific^21,26^. The defined HBGA phenotypes and huNoV binding features of these two HIOs have laid a solid basis for further characterization of huNoV-host interaction and huNoV infection.

Based on the recent success of huNoV replication in HIEs^19^, we performed an initial attempt to replicate huNoVs in the HIOs and obtained promising results on potential usefulness of HIOs to study huNoV replication. After inoculation with GII.4 huNoV-positive stool filtrates to HIOs with matched secretor HBGA types, we were able to detect 1) huNoV RNAs by RT-PCR, 2) huNoV capsid antigens by immunostaining followed by fluorescent microscopy, 3) inclusion bodies with typical viral particles by EM, and 4) immunogold signals by EM indicating presence of huNoVs. In addition, we were able to detect viral RNAs by RT-PCR after 10 passages of the original inoculum in fresh HIOs, excluding the possibility of detecting non-replicating residual huNoVs from the original inoculum.

However, we also noted that our HIO model at current set up produced only small amount of replicating viruses compared with the HIE approach that usually increases viral titers for several logs^19^. The major reason for this difference may be that the HIE approach provides much larger numbers of enterocytes for huNoV infection than our HIO model. In our study, huNoV stool filtrate was inoculated to a single or a few cut HIO in Matrigel that contain very limited numbers of differentiated enterocytes, whereas the HIE method cultured monolayer cells from multiple HIEs after trypsinization, providing much more differentiated enterocytes for huNoV infection. However, our study did not measure huNoV replication in immature HIO for comparison and thus, the effects of HIO maturity or differentiation level on norovirus replication remain to be defined. Secondly, the status of the enterocytes from HIEs may differ from those in HIOs, in other words, enterocytes from HIEs may be more sensitive to huNoV infection compared with those from HIOs. While HIOs developed from embryonic PSCs that needed more induction steps by more grow factors, HIES derived directly from the intestinal stem cells that constitute the crypts of the intestinal epithelia. As a result, HIEs can be easily cultured from the isolated intestinal crypts, while HIOs need to go through a long process from an embryonic stem cell line.

However, the long development process of an HIO may provide an opportunity to study the developmentally regulated dynamics of HBGA expression. HBGA expression is known to be age-regulated in humans^27^, which may further affect the host susceptibility to HBGA-recognized pathogens, such as huNoVs and rotaviruses. In this regard, the long HIO development process that goes through the endoderm and hindgut spheroid stages may offer a unique model system to study the age-regulated dynamics of HBGA expressions and distributions. This in turn provides a useful tool to study age-specific HBGA-huNoV/rotavirus interaction, as well as age-specific susceptibility to the two enteric viruses causing gastroenteritis.

## Materials and Methods

### HIO development and maintenance

PSCs were provided by the Pluripotent Stem Cell and Organoid Core (https://research.cchmc.org/stemcell/overview) at Cincinnati Children’s Hospital Medical Center. The development and maintenance of HIOs were performed according to the protocols provided by the same core, as well as the methods described elsewhere^1–3^.

### Electron microscopy (EM) and immunogold staining

HIO samples were fixed with 4% formaldehyde and embedded in gelatin capsule filled with L.R. White. Trimmed EM blocks were cut in ultrathin sections at 60–90 nm. Sections were placed on formvar-carbon coated nickel grids for EM observation after contrast staining with uranyl acetate and lead citrate. For immunogold staining, sections on grids were rinsed with a large drop of TBS-tween, followed by blocking with 3% normal goat serum. The sections were then incubated on droplets of primary antibody specific to GII.4 huNoVs^21,26^ for 10 hours at 4 °C. After rinsing on large droplets of TBS-Tween, the sections were incubated on droplets of biotinylated secondary antibody, followed by incubation with Streptavidin-Gold conjugates. The sections were then post-fixed on droplets of 2% glutaraldehyde and then rinse in TBS-Tween. Contrast staining was performed using uranyl acetate and lead citrate. The stained sections were observed under EM.

### Immunofluorescent microscopy and confocal microscopy

Immunofluorescent microscopy was used to 1) identify cellular compositions of HIO epithelia, 2) elucidate the HBGA expression profiles of the HIOs, 3) determine the specific interaction between huNoVs and HIOs, and 4) detect the replicated huNoVs in the HIOs. For identification of cellular compositions of HIO epithelia, monoclonal antibodies (Mabs) specific to mucin 2 (biomarker of goblet cells) (Santa Cruz Biotechnology, 1:200), lysozyme (biomarker of Paneth cells) (Zymed Laboratories, 1:1000), and chromogranin A (biomarker of endocrine cells) (ImmunoStar, 1:1000) were used. HIO section slides were incubated with these primary antibodies overnight at 4 °C), followed by incubations with Alexa Fluor-labeled secondary antibodies (Invitrogen, 1: 200) at room temperature for 2 hours. For elucidation of the HBGA expression profiles of HIOs, Mabs specific to H1, H2, Le^a^, Le^b^, Le^x^, Le^y^, and sialyl Le^x^ (BioLegend, San Dego, CA) were used. HIO section slides were incubated with these Mabs (1:100) for overnight at 4 °C, followed by incubations with Alexa Fluor-labeled secondary antibodies (goat anti mouse IgG, Invitrogen, 1: 100) at room temperature for 2 hours. For determination of the interactions between HIOs and various huNoV capsids, HIO section slides were incubated with huNoV VLPs^21,26^ at 30 ng/mL for 2 hours. The HIO-bound VLPs were detected by hyperimmune serum against various huNoV VLPs^21,26^, followed by incubations with fluorescently labeled secondary antibody (goat anti rabbit IgG, Invitrogen, 1: 300). For detection of replicated GII.4 huNoVs in the HIOs, hyperimmune serum against GII.4 huNoV VLPs^21,26^ and the secondly antibody, as well as the same procedure above were used. Nuclei of HIOs were stained using TO-PRO-3 stain (Thermo-Fisher Scientific). All VLP used in this study were from our lab stock that were made in our previous studies^21,26^.

### Viral RNA extract, reverse-transcript (RT) PCR, and DNA sequencing

HuNoV RNAs were extracted from stool and HIO samples using the RNeasy Plus Kits (Qiagen). HuNoV RNAs were reverse transcribed into cDNAs using cDNA Synthesis Kits (Qiagen). PCR was used to detect huNoV genome using primer pair 289 and 290 specific to huNoV genomic region encoding the RNA dependent RNA polymerase^28^. The RT-PCR products were purified and cloned into pGEM-T Easy vector (Promega) for sequencing. DNA sequencing was performed by the DNA Sequencing and Genotyping Core at Cincinnati Children Hospital Medical Center.

### Sequence-specific primer-directed PCR (SSP-PCR)

SSP-PCR was performed to test three predominant nonsense mutations (A358T, G428A, and C571T) in the gene encoding α 1,2-fucosyltransferase (FUT2) that is a key enzyme to catalyze the formation of H antigens. DNA was extracted from buccal cells in saliva samples using the Oragene DNA collection Kit (DNA Genotek). Primers with specific 3′-end residues to wild type (W) or mutant (M) nucleotide of the three *FUT2* mutation sites, respectively, were synthesized (Table 1). An antisense primer (Table 1) was paired with wild-type and mutated sense primers to amplify the corresponding gene fragments by SSP-PCRs. The gene encoding human growth hormone (hGH) was served as an internal PCR control. All primer sequences and the expected PCR product sized are listed in Table 1. All SSP-PCRs were carried out in the following program: 95 °C, 2 min, then 10 cycles of 95 °C 20 sec, 66 °C 1 min, 70 °C 1 min; followed by 20 cycles of 95 °C 20 sec, 62 °C 1 min, 72 °C 1 min; and a final extension at 72 °C for 5 min.

### HuNoV filtrates and HIO inoculation

Stool filtrates were prepared from GII.4 huNoV positive stool samples that were collected from a previous human challenge study^14^. PBS solution was mixed with stool samples (10:1, V:V) and vortexed, followed by a centrifugation at 10,000 rpm for 5 min using a benchtop centrifuge. Clear supernatant was filtrated through 0.45 µm and then 0.22 µm filters to remove bacterial pathogens. The viral stock contains ~3.42 × 10^5^ RNA copies/mL. For huNoV inoculation, HIOs were cut in half and a further diluted viral stock (1:500) in culture media (DMEM) without fetal bovine serum (FBS) were added to the cut HIOs and incubated at 37 °C for overnight (16 hours). Then the inoculated HIOs were washed with DMEM medium without FBS 5 times and cultured in DMEM medium with 10% FBS for further four days. Inactivated viruses after boiling for 10 min were used as negative controls.

### Multiple passages of huNoV-infected HIOs

To confirm that huNoVs can replicate in the HIOs, multiple passages of huNoV infected HIOs were performed. Three to four days after inoculation, the inoculated HIOs were homogenized to release viruses from the cells. After centrifugation the supernatant of the homogenized HIOs was used for huNoV RNA detection via RT PCR, as well as for inoculation to fresh HIOs. This was repeated for ten times.
